# *In vitro* antibacterial activities and molecular characterization of bacterial species isolated from farmlands against selected pathogens

**DOI:** 10.1016/j.btre.2020.e00513

**Published:** 2020-08-09

**Authors:** Ismail B. Onajobi, Esther O. Idowu, Jamiu O. Adeyemi, Oyindamola J. Samson, Peter I. Ogunyinka, Obasola E. Fagade

**Affiliations:** a Department of Microbiology, Faculty of Science, Olabisi Onabanjo University, Ago-Iwoye, Ogun State, Nigeria; b Department of Mathematical Sciences, Faculty of Science, Olabisi Onabanjo University, Ago-Iwoye, Ogun State, Nigeria; c Department of Microbiology, Faculty of Science, University of Ibadan, Nigeria

**Keywords:** Analysis, Antibacterial, Electrophoresis, Identification, Molecular, Pathogenic, Phylogenetic

## Abstract

This study aims to screen bacterial isolates from Olabisi Onabanjo University Farmland for antibacterial activity against pathogenic microorganisms. Agar well diffusion method was used. Isolates were identified molecularly. Chi-square test revealed significant association between isolates, antibacterial activity with likelihood *p*-value = 0.000 and 5% significant level. Six among thirty-five isolates exhibited antibacterial activity against the test pathogenic species. A greater antibacterial activity (50 % inhibition) was observed in *Lysinibacillus sphearicus* strain PRE16. It inhibited the growth of *Bacillus subtilis, Staphylococcus aureus* and *Escherichia coli* by 23.00 ± 2.00, 18.00 ± 2.00 and 20.00 ± 4.00 respectively. DNA sequencing revealed antagonist isolates as *Bacillus* sp. BCN2, *Brochothrix thermosphacta* strain P30C4, *Bacillus aryabhattai* strain KNUC205, *Alcaligenes faecalis* strain KEM24*, Bacillus arsenicus* strain CSD05 and *Lysinibacillus sphaericus* strain PRE16. Phylogenetic analysis revealed close relatedness of most isolates with *Bacillus* species strains. These strains are suggested to be effective for the discovery of new antibacterial agents.

## Highlights

• Screening bacterial isolates for antagonistic potential.
• Molecular methods for identification of isolates.
• Six potent antibacterial isolates against the test pathogenic species.
• *Pseudomonas* was susceptible to antagonizing strains.
• Necessity for new effective antibacterial formulation.

## 1 Introduction

Since ancient times, humans have been faced with innumerable number of diseases. Most of these diseases are caused by microorganisms [[1], [2], [3], [4]]. In the treatment of diseases, various therapeutic measures have been put in place. Since the discovery of a microorganism with antibacterial activity in 1928, there have been incessant studies and use of microbes for the production of antimicrobial compounds against disease etiological agents [5].

Most of these microorganisms with antimicrobial potentials have been isolated in various habitats. The most suitable habitat of microorganisms as reported by [6] is the soil. It is a loose natural component consisting of mixtures of organic components and minerals [7].

Soil-dwelling microorganisms with antimicrobial potentials have been reported by various research findings. [8,9] reported antimicrobial activities of some fungal species such as *Penicillium* spp.*, Aspergillus* spp., *Ganoderma lucidium* and *Absidia corymbifera* against the growth of *Candida* species*, Escherichia coli, Pseudomonas aeruginosa* and *Staphylococcus aureus*. According to [10], these antimicrobial activities were as a result of the production of some compounds which include nigerazine B, tensidol A and so on. Research findings have also reported the antimicrobial potential of bacterial species such as *Escherichia coli*, *Pseudomonas* species, *Actinobacteria* and so on [[11], [12], [13]]. Etiological agents of human and plant diseases which include *Salmonella* species, *Clostridium difficile*, *Fusarium verticillioides* and *Phaeomoniella chlamydospora*, are susceptible to their antimicrobial effects [14,15]. They also produce some antimicrobial compounds such as hydrogen cyanide, monoacetylphloroglucinol (MAPG) and microcin for growth inhibition. *Actinobacteria* are widely known for antimicrobial activities against an enormous number of pathogens [16,17]. They produce over 60 % of antimicrobial compounds used around the world today. Two common genera of these bacteria widely studied for antimicrobial potential include *Streptomyces* and *Micromonospora*. They produce antibacterial agents belonging to the class of antibiotics which include macrolides, β–lactams, aminoglycosides, glycopeptides and so on [[18], [19], [20]]. *Bacillus* species are also known for antibacterial potential against the growth of common human and plant pathogens such as *Staphylococcus aureus, Escherichia coli,* and *Rhizoctonia solani* [7,8,21,22]. They are frequently studied for the production of antimicrobial compounds of varying structures and chemical properties. Such compound includes bacteriocin [23].

Most of these antimicrobial compounds have become less effective day by day due to the emergence and development of resistance to these compounds by the disease etiological agents [24]. This makes most diseases very difficult to treat [3], propelling the need to do more research findings and exploring farmland soil which could be a potential environment to discover microorganisms with appreciable antibacterial potential [9]. This study, therefore aims to investigate the antibacterial potential of bacterial isolates from Olabisi Onabanjo University farmland.

## 2 Materials and methods

### 2.1 Collection of soil samples

**Fig. 1:**
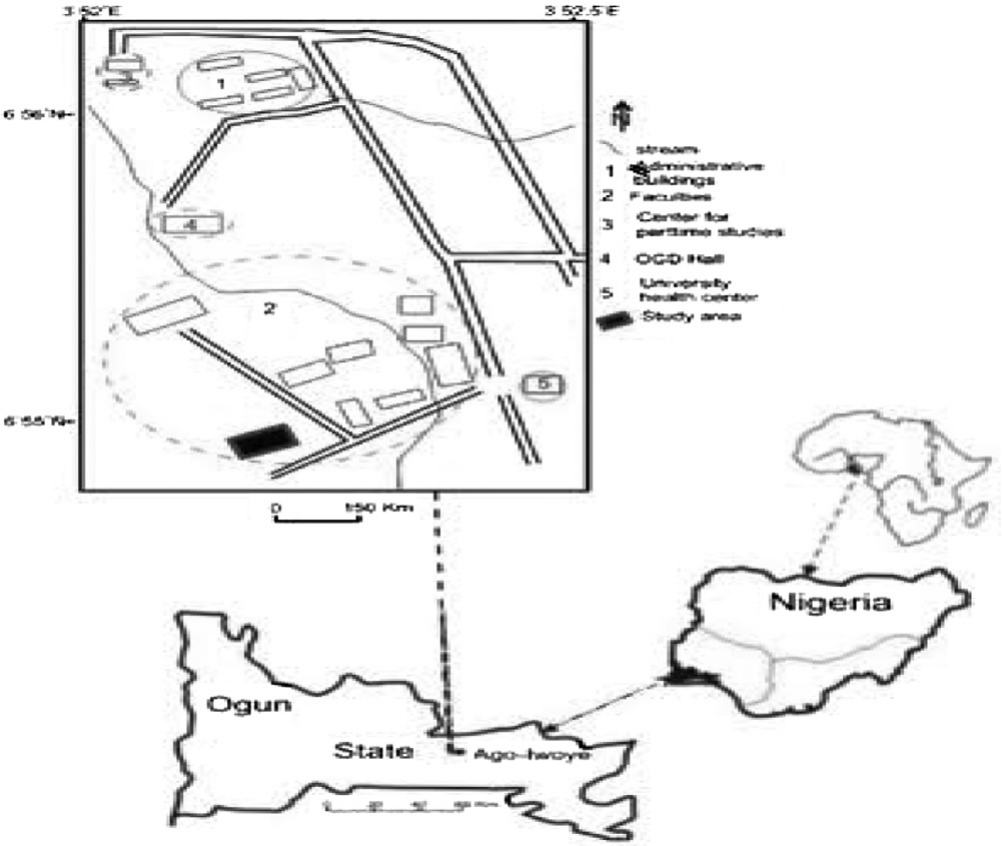
Map of sample location with the google map Uniform locator https://goo.gl/maps/qwbkbkMcvPZ37Hyk7.

The soil samples (50 g) were randomly collected from farmland in Olabisi Onabanjo University, Ago-Iwoye, Ogun State, Nigeria around maize and pineapple plantation. The soil top layer was collected aseptically in a container. Soil samples were taken to the laboratory immediately after collection (Fig. 1).

### 2.2 Source of test organisms

The test organisms were *Escherichia coli, Staphylococcus aureus, Klebsiella pneumonia*, *Bacillus subtilis* and *Pseudomonas aeruginosa.* Stock culture of these organisms was obtained from Nigerian Institute of Medical Research (NIMR). The cultures were sub-cultured on solidified nutrient agar prior to use [25].

### 2.3 Isolation of organisms

A series of dilution were carried out in order to obtain numerable number of colony [26]. These dilutions were evenly spread on the solidified nutrient agar (NA) and incubated for 2 days at 30 °C. The resulted colonies were enumerated and subjected into the subsequent purification and subculture, on the NA medium [10,27].

### 2.4 Standardization of the test organisms

The 0.5 McFarland Standard prepared was used to standardize the test organisms [28]. The stock cultures were firstly purified by sub-culturing on solidified nutrient agar and incubating for 24 h at 37 °C. They were then inoculated into sterile nutrient broth and incubated for 4 h at 37 °C. The turbidity of the bacterial suspension was compared with the turbidity of the prepared standard by placing both tubes near a white sheet of paper having black stripes to enhance easy detection of turbidity differences [11].

### 2.5 Preparation of cell free supernatant (CFS)

The pure isolates were inoculated into nutrient broth and incubated 48 h at 30 °C. The CFS was obtained by centrifuging the broth culture at 5000 g force for 15 min and used immediately [12,29].

### 2.6 Screening of isolates for antibacterial activities

The antibacterial activity of the isolates CFS was determined by the employment of the well diffusion method as described by [30]. Using a sterile swab stick, standardized culture of test organisms was streaked evenly on the entire surface of the solidified agar plate. The plates were allowed to dry for 15 min. A sterile cork borer hole of 9 mm in diameter was used to create wells on the solidified agar containing streaked organisms. In each well, 0.1 mL cell free supernatant culture of isolates was dispensed. The plates were incubated for 24 h at 30 °C. The diameters of inhibition were recorded in millimeter for each isolates [31].

### 2.7 Identification of the antibacterial potent isolates

The isolates that displayed antibacterial activities were characterized based on their biochemical characteristics. This was achieved by carrying out gram staining, catalase test, urease test and starch hydrolysis [32]. Molecular characterization of these isolates was also performed [33].

Gram staining was carried out to identify isolates if they are gram-positive or gram-negative. The isolates were smeared on a clean, grease-free and dry glass slide. It was allowed to air-dry completely and then heat fixed by passing through the flame 3 times. It was allowed to cool for 15 min. Crystal violet stain (primary stain), Lugol’s iodine, Acetone-alcohol decolourizer and Safranin (counter-stain) were dispensed on the smear. Each reagent was allowed to stay on the smear for 60 s, followed by subsequent rinsing with clean water. Each stained smear was placed on a draining rack to air dry. The smear was then observed microscopically first with 40x objective to check the staining and secondly with an oil immersion objective to report the cellular morphology of the isolates and their ability to retain the primary stain [34]. Colonies were tested for the possession of catalase enzyme, whereby a pure colony was placed on a surface of clean, dry glass slide using a loop. A drop of 3% H_2_O_2_ was placed onto the slide and mixed [35]. The production of bubbles was checked for.

For urease test, urease medium in test tubes was inoculated with a loopful of pure culture of the isolates. The cap was loosely fixed on the tubes. The test tubes were incubated at 35 °C in ambient air for 18–24 hours. Agar slant was observed for colour change.

Isolates were inoculated on the starch agar to test for their ability to hydrolyse starch. Plates were incubated for 24 h at 37 °C. After growths were observed, plates were covered with iodine. The productions of clear zones around growths were checked for, after 10 min [36].

### 2.8 Molecular identification

The antagonist isolates were inoculated into sterile nutrient broth and incubated at 37 °C for 24 h. The resulting bacterial cells were suspended and re-suspended into 200 μL of water [37].

### 2.9 DNA extraction

Fifty milligram (50 mg) (wet weight) of the bacterial cells that have been re-suspended were dispensed into a ZR Bashing™Lyses Tube. Lysis Solution (750 μL) was added. This tube was secured in a bead beater fitted with 2 mL tube holder assembly. The Mixture was processed at maximum speed for 5 min [38]. This was subjected to centrifugation using a micro centrifuge at >10,000 x g for 1 min. The resulting supernatant (400 μL) was transferred to a Zymo-Spin™ IV Spin Filter (orange top) in a collection tube and was centrifuged at 7000 x g for 1 min. The base of this Zymo-Spin ™ Spin filter was snapped off before use. Bacterial DNA Binding Buffer (1,200 μL) was added to the filtrate in the collection tube.

The mixture (800 μL) was transferred to a Zymo-Spin™ IIC Column in a collection tube and centrifuge at 10,000 x g for 1 min. The flow was discarded through the collection tube and 800 μL of the mixture (1,200 μL of Bacterial DNA Binding Buffer and the filtrate in the collection tube from the initial centrifugation of 400 μL supernatant) was again transferred to the Zymo-Spin™ IIC Column in a collection tube. This was centrifuged at 10,000 x g for 1 min. Two hundred (200) μl DNA Pre-Wash Buffer was then added to the Zymo-Spin™ IIC Column in a new collection tube and centrifuge at 10,000 x g for 1 min. Bacterial DNA Wash Buffer (500 μL) was added to the Zymo-Spin™ IIC Column and centrifuge at 10,000 x g for 1 min. The Zymo-Spin™ IIC Column was transferred to a clean 1.5 mL Microcentrifuge tube. DNA Elution Buffer (100 μL) was added directly to the column matrix. This was centrifuged at 10,000 x *g* for 30 s to elute the DNA [37].

### 2.10 DNA amplification

The extracted DNA, which is about 900 bp was utilized as a template for 16S rRNA gene amplification. This gene was amplified using the forward and reverse primers (16SF: GTGCCAGCAGCCGCGCTAA and 16SR: AGACCCGGGAACGTATTCAC) of about 0.5 μL. Taq 5 u/ul (0.1 μL), 10x PCR buffer (1.0 μL), 25 mM Mgcl_2_ (0.1 μL), 0.1 μL (DMSO), 2.5 Mm DNTPs (0.8 μL), 10 ng/μl DNA (2.0 μL) and 3.1 μL distilled water was used. Thermal cycler of Eppendorf 96AG, model 2231 was utilized in the amplification process. PCR conditions include initial denaturation at 94 °C for 5 min. Another denaturation at 94 °C for 30 s. Annealing was at 56 °C for 30 s. Initial extension was at 72 °C for 45 s. Final extension was at 72 °C for 7 min. The Holding temperature of the process was 10 °C. All the three processes (Denaturation, Annealing and Extension of primers) were repeated 36 times giving rise to 36 cycles [38].

### 2.11 PCR product purification

The PCR products, also known as amplicons were purified by adding absolute ethanol (2 volumes) to the products. This mixture was incubated at room temperature for 15 min and spin down at 10,000 rpm for 15 min. Supernatant was decant and also spin down at 10,000 g force for 15 min. Two (2) volumes (40 μL) of 70 % ethanol were added and supernatant was decant and air dry. Ultrapure water (10 μL) was added and amplicons were observed on 1.0 % agarose [39].

### 2.12 Agarose gel electrophoresis

The amplicons (resulting amplification products) from these processes were loaded on 1.0 % agarose gel. This was prepared by dissolving 1.0 g of agarose powder into 100 mL of 20x stock solutions SB buffer. SB buffer was prepared by dissolving 8 g of NaOH into 45 g Boric acid in 1 l of distilled water. 250 mL of this solution was added to 4.75 l of water. The gel solution was evenly mixed by stirring and placed in microwave for 2 min for homogeinity and to produce solidified gel when cooled [40].

**Fig. 2:**
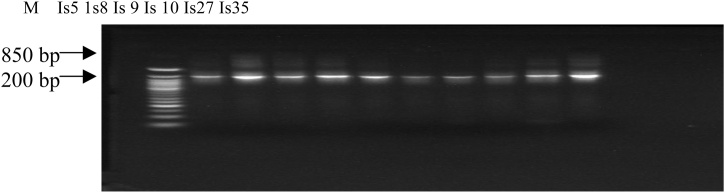
Gel image of PCR purified DNA product. **Key:** M = Molecular marker 900bp molecular size; Is5, 1 s 8, Is 9, Is 10, Is 27 and Is 35= Isolate 5, 8, 9, 10, 27 and 35 respectively.

The gel was left to cool and dispensed into gel plate having combs placed in the gel caster. This was left to solidify. Solidified gel was placed in the electrophosis chamber. 3 μL of loading dye was added to 7 μL of DNA and was dispensed into holes created by the combs. 3 μL ethidium bromide was incorporated into the gel to enhance easy visualization of the DNA under ultraviolet light. All of these were subjected to electrophoresis for 45 min at 80 V [41]. The ladder used was hyper ladder 1 from Bioloine (Fig. 2).

### 2.13 Data processing of the isolates’ 16S rRNA sequences

The DNA was loaded on the Applied Biosystem 3500 genetic analyser from Applied Biosystems to give the sequences [42].

The BioEdit sequence alignment editor was used for editing and aligning the sequences. These edited sequences were exported and saved in FASTA format. The 16S rRNA gene sequences were compared with sequences in the Gene Bank database using basic local alignment search tool (BLAST) in the National Centre for Biotechnology Information (NCBI) to check for similarities with other existing sequences.

Accession number was assigned to each related sequence by the NCBI gene bank. The isolates’ 16S rRNA gene sequences with high similarities to the sequences were compared for relatedness using Molecular Evolutionary Genetics Analysis (MEGA) version 5.05 [43]. This was used to construct a maximum likelihood phylogenetic tree.

### 2.14 Molecular phylogenetic analysis

The evolutionary history of isolates was inferred by using the Maximum Likelihood method based on the Tamura-Nei model [44]. The tree with the highest log likelihood (-695.55) was shown. Initial tree(s) for the heuristic search were obtained automatically by applying Neighbor-Join and BioNJ algorithms to a matrix of pairwise distances estimated using the Maximum Composite Likelihood (MCL) approach, and then selecting the topology with superior log likelihood value. The analysis involved 5 nucleotide sequences. All positions containing gaps and missing data were eliminated. There were a total of 511 positions in the final dataset. Evolutionary analyses were conducted in Molecular Evolutionary Genetics Analysis (MEGA) version 5.05 [45].

### 2.15 Statistical analysis

Chi-Square of independence test was used to test for significant association between the inhibition and the isolates [76].

## 3 Results

Table 1 shows the antibacterial activities of thirty-five (35) isolates against common human pathogens which include *Staphylococcus aureus, Escherichia coli, Klebsiella pneumoniae, Bacillus subtilis* and *Pseudomonas aeruginosa*. Six (6) isolates showed antibacterial activities. Is 5, 1 s 8, Is 9, Is 10 and Is 27 inhibited the growth of *Pseudomonas aeruginosa* with a zone of average inhibitions of 15.00 ± 1.00, 11.00 ± 1.73, 15.30 ± 1.15, 10.00 ± 1.15 and 18.00 ± 2.00 mm, respectively.

**Table 1:** Antibacterial activities of Isolates in millimeter (mm).

Isolates code	*Bacillus subtilis*	*Staphylococcus aureus*	1 *Klebsiella* 2 *pneumoniae*	*Escherichia coli*	*Pseudomonas aeruginosa*
	**Zone of Inhibition (mm)**				
Is 1	–	–	–	–	–
Is 2	–	–	–	–	–
Is 3	–	–	–	–	–
Is 4	–	–	–	–	–
Is 5	–	–	–	–	–
Is 6	–	–	–	–	–
Is 7	–	–	–	–	–
Is 8	–	–	–	–	11.00 ± 1.73
Is 9	–	–	–	–	15.30 ± 1.15
Is 10	–	–	–	–	10.00 ± 1.15
Is 11	–	–	–	–	–
Is 12	–	–	–	–	–
Is 13	–	–	–	–	–
Is 14	–	–	–	–	–
Is 15	–	–	–	–	–
Is 16	–	–	–	–	–
Is 17	–	–	–	–	–
Is 18	–	–	–	–	–
Is 19	–	–	–	–	–
Is 20	–	–	–	–	–
Is 21	–	–	–	–	–
Is 22	–	–	–	–	–
Is 23	–	–	–	–	–
Is 24	–	–	–	–	–
Is 25	–	–	–	–	–
Is 26	–	–	–	–	–
Is 27	–	–	–	17.30 ± 2.51	18.00 ± 2.00
Is 28	–	–	–	–	–
Is 29	–	–	–	–	–
Is 30	–	–	–	–	–
Is 31	–	–	–	–	–
Is 32	–	–	–	–	–
Is 33	–		–	–	–
Is 34	–	–	–	–	–
Is 35	23.00 ± 2.00	18.00 ± 2.00	–	20.00 ± 4.00	–

**Keys**: Is = Isolate; - = No reaction; + = Reaction; ZOI (mm) = Zone of Inhibition measured in millimeter. Values were mean of three determinations ± S.E.M.

Is 35 inhibited the growth of *Bacillus subtilis* and *Staphylococcus aureus* with a zone of inhibition 23.00 ± 2.50 and 18.00 ± 2.00 mm respectively. While *Escherichia coli* was susceptible to Is 27 by 17.30 ± 2.51 zone of inhibition in mm. Is 35 exhibited highest antibacterial activities among the six active isolates. It exhibited the growth of *Bacillus subtilis, Escherichia coli* and *Staphylococcus aureus* with a zone of inhibition 23.00 ± 2.00, 20.00 ± 4.00 and 18.00 ± 2.00 mm respectively. *Klebsiella pneumoniae* was resistant to all the active antibacterial producing isolates (Table 2).

**Table 2:** Observed and Expected inhibition values.

			Reaction		Total
			No	Yes	
Isolate Codes1	isolation A	Count	145	0	145
		Expected Count	137.5	7.5	145.0
	isolation B	Count	21	9	30
		Expected Count	28.5	1.5	30.0
Total		Count	166	9	175
		Expected Count	166.0	9.0	175.0

Chi-square test result (in Table 3, Table 4) tested for significant association between the inhibitions and the isolates. The null hypothesis of no significant association was rejected based on the likelihood ratio *p-value* = 0.000 at 5% significance level. Hence, it was concluded that the isolate had significant association with the inhibition. It was, also, affirmed that there was significant and moderate [76] association of 51.2 % (Phi value in Table 5, Table 6). The antagonism percentage displayed by antagonistic isolates as shown in Table 7, reveals Is 35 having 50 % inhibitory activity. 20 % inhibitory activity was observed in Is 27, which inhibits BS, SA and EC. Is 5, 8, 9 and 10 exhibit inhibitory percentage of 16 %.

**Table 3:** Chi-Square test of association between the 35 isolates and the inhibition.

	Value	Df	Asymp. Sig. (2-sided)	Exact Sig. (2-sided)	Exact Sig. (1-sided)
Pearson Chi-Square	45.858^a^	1	.000		
Continuity Correction^b^	39.915	1	.000		
Likelihood Ratio	34.293	1	.000		
Fisher's Exact Test				.000	.000
N of Valid Cases	175				

a. 1 cells (25.0 %) have expected count less than 5. The minimum expected count is 1.54.

b. Computed only for a 2 × 2 table.

**Note:***p*-value (Asymp. Sig.) for likelihood ratio is 0.000. The null hypothesis is rejected at 5% significant level.

**Table 4:** Test of Independence/relationship strength (Symmetric Measures).

		Value	Approx. Sig.
Nominal by Nominal	Phi	.512	.000
	Cramer's V	.512	.000
N of Valid Cases		175

**Table 5:** Biochemical characteristics of Antagonistic Isolates.

Isolates code	Morphology	Gram stain	Catalase	Urease	Starch Hydrolysis
Is 5	Rod	+	+	–	+
Is 8	Rod	+	+	–	–
Is 9	Rod	+	+	–	+
Is 10	Rod	–	+	+	–
Is 27	Rod	+	+	–	+
Is 35	Rod	+	+	+	–

**Keys**: Is = Isolate; - = No reaction; + = Reaction.

**Table 6:** Screening for antagonism among experimental bacterial isolates.

Antagonistic isolates	Number of positive records	Antagonized isolates	Antagonisms%
Is 5	1	PA	16
Is 8	1	PA	16
Is 9	1	PA	16
Is 10	1	PA	16
Is 27	2	PA & EC	33
Is 35	3	BS, SA & EC	50

**Keys**: PA – *Pseudomonas aeruginosa,* EC - *Escherichia coli,* BS - *Bacillus substilis,* SA - *Staphylococcus aureus*, Is – Isolates.

**Table 7:** Molecular identification of Isolates based on 16S rRNA sequencing.

**Isolate code**	**Species identify**	**Strain**	**Accession number**	**Similarity %**
Is 5	*Bacillus* sp.	BCN2	**JX045721.1**	82 %
Is 8	*Brochothrix thermosphacta*	P30C4	**MH000378.1**	86 %
Is 9	*Bacillus aryabhattai strain*	KNUC205	**JN051485.1**	93 %
Is 10	*Alkaligenes faecalis*	KEM24	**MK595710.1**	99 %
Is 27	*Bacillus arsenicus*	CSD05	**HM100220.1**	83 %
Is 35	*Lysinibacillus sphaericus*	PRE16	**EU880531.1**	99 %

**Key**: Is = Isolate.

The biochemical characteristics of isolates displaying antibacterial activities are shown in Table 5. All isolates are rod shaped and catalase positive. All except Is 10 are gram positive. Is 35 is the only urease producing isolate. Is 5, Is 9 and Is 27 are starch hydrolyzing isolates, while Is 8, Is 9 and Is 35 are non-starch hydrolyzing isolates. The probable bacteria identified are *Streptomyces, Bacillus, Rhizobium* and *Norcadia* spp.

Molecular sequencing of the 16S rRNA gene of the isolates as shown in Table 3 revealed the identified strains of the isolates, their accession number and similarity percentage. Is 5, 1 s 8,Is 9,Is 10 and Is 27 have a similar percentage of 82 %, 86 %, 93 %, 99 %, 83 % and 99 % with *Bacillus* sp. BCN2, *Brochothrix thermosphacta* strain P30C4, *Bacillus aryabhattai* strain KNUC205, *Alcaligenes faecalis* strain KEM24*, Bacillus arsenicus* strain CSD05 and *Lysinibacillus sphaericus* strain PRE16 with accession number **JX045721.1, MH000378.1, JN051485.1, MK595710.1, HM100220.1** and **EU880531.1** respectively.

**Plate 1:**
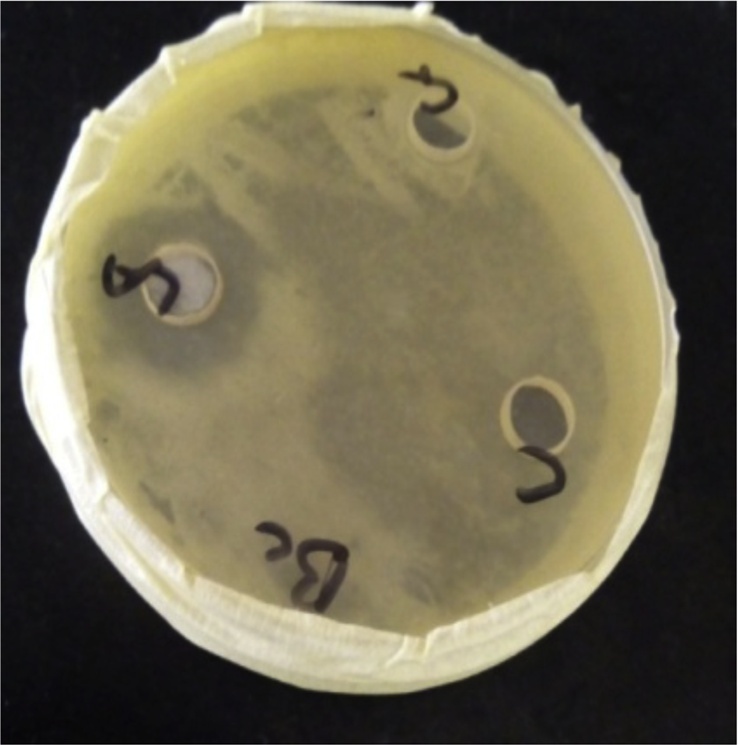
Antibacterial activity of some isolates against *Bacillus subtilis*.

**Plate 2:**
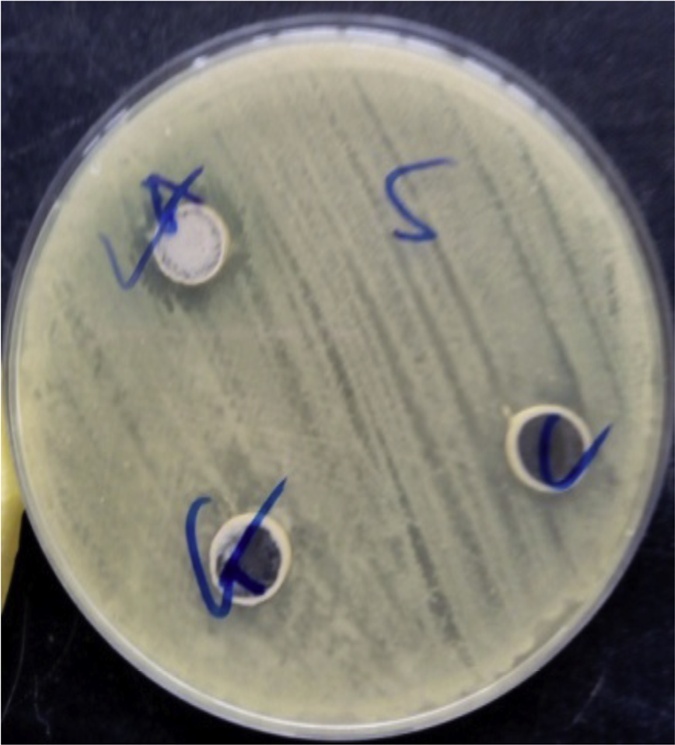
Antibacterial activity of some isolates against *Staphylococcus aureus*.

**Plate 3:**
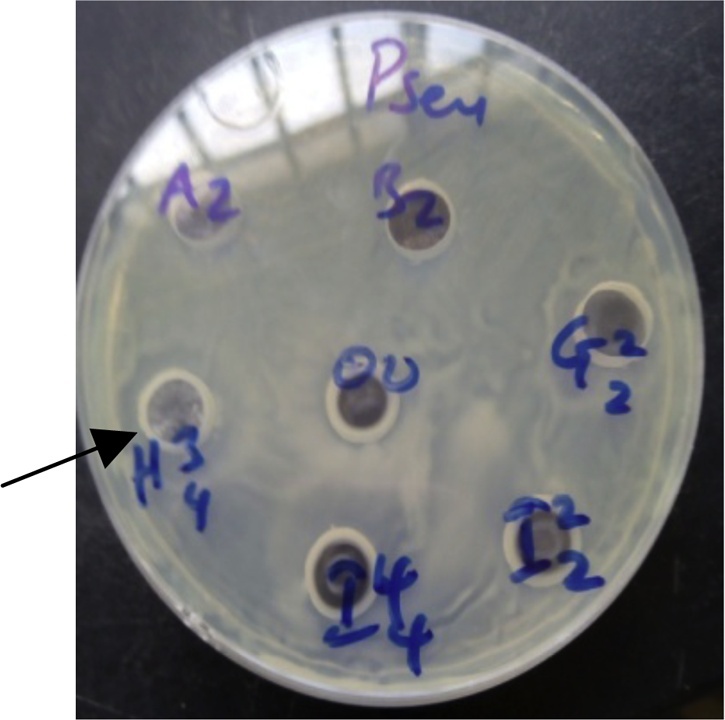
Antibacterial activity of some isolates against *Pseudomonas aeruginosa*.

Plate 1, Plate 2, and Plate 3 reveal antibacterial activities of some isolates against *Staphylococcus aureus, Bacillus subtilis* and *Pseudomonas aeruginosa*.

**Fig. 3:**
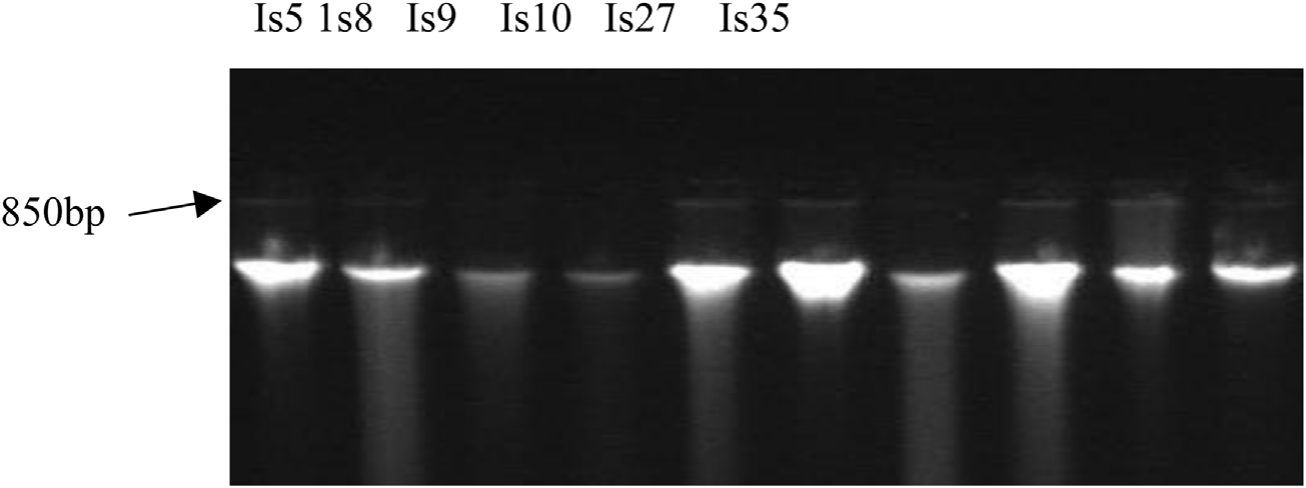
Full gel image of PCR purified DNA product. **Key:** Is5, 1 s8, Is9, Is10, Is27 and Is 35 = Isolate 5, 8, 9, 10, 27 and 35 respectively

**Fig. 4:**
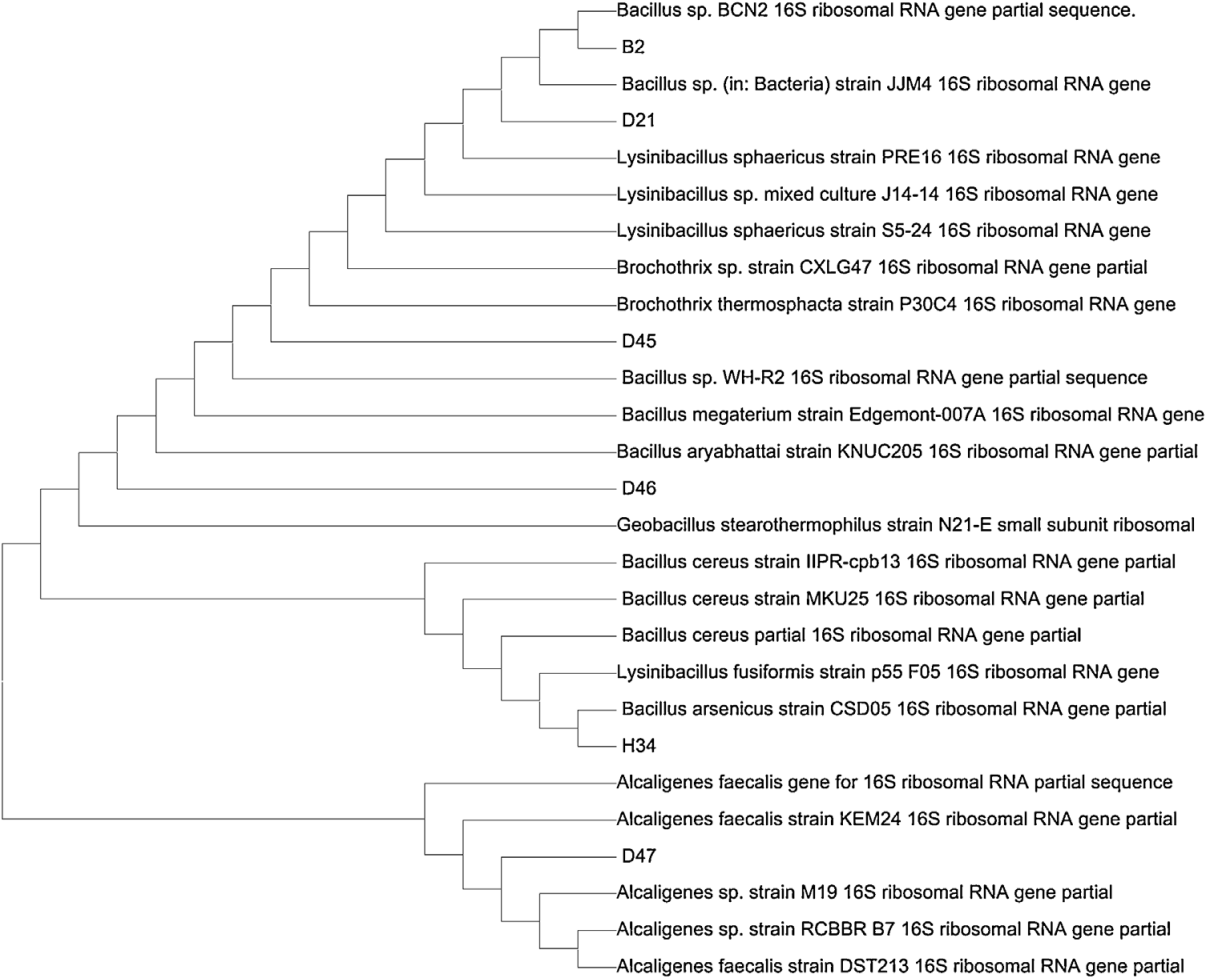
Phylogenetic tree constructed using MEGA 5.05 based on homologous sequences of antibacterial potent isolates. **Keys:** B2= Isolate (Is) 5; D21= Isolate (Is) 35; D45= Isolate (Is) 8; D46= Isolate (Is) 9; H34= Isolate (Is) 27; D47= Isolate (Is) 10

Fig. 3, Fig. 4 show the gel image of the PCR purified DNA product of the isolates revealing their DNA size when compared with a molecular marker which serves as a reference marker. The DNA sizes are expressed in base pair. The molecular marker is around 900 bp. The six isolates (Is 5, 1 s 8, Is 9, Is 10 and Is 27) are around 850 bp. A phylogenetic tree revealing the evolutionary relationship between the DNA sequences of the isolates and the bacteria strains in the NCBI DNA database is shown in Fig. 5. All the isolates are closely related with *Bacilli* species except Is 10. Is 5 (B2), Is 8 (D45), Is 9 (D46), Is 10 (H34), Is 27 (H34) and Is 35 (D21) form clusters with *Bacillus* sp. BCN2, *Brochothrix thermosphacta* strain P30C4, *Bacillus aryabhattai* strain KNUC205, *Alcaligenes faecalis* strain KEM24*, Bacillus arsenicus* strain CSD05 and *Lysinibacillus sphaericus* strain PRE16 respectively. Is 5 (B2) and ls 27 (H34) form clade with *Bacillus* sp. BCN2 and *Bacillus arsenicus* strain CSD05 respectively.

## 4 Discussion

Farmland contains enormous microorganisms, including those with unique characteristics due to its nutritious constituents. These include organic matter, moisture, essential elemental components like nitrogen, phosphorus, potassium and so on. This facilitates their growth, multiplication and viability [46,47]. Among these unique characteristics include antibacterial potentials. According to [48], these potentials are as a result of metabolites they produced. Cell free supernatant (CFS) of bacteria contain these metabolites, and has been recently used by researchers to elicit antimicrobial effects on pathogens. [49,50], used the CFS of *Bacillus* species to inhibit the growth of bacterial pathogens. [51], reported the antimicrobial activities of various antimicrobial compounds which include lactic acid, acetic acid and H_2_O_2_ in the CSF of *Weissella cibaria* against malodor inducing bacteria like *Fusobacterium nucleatum, Porphyromonas gingivalis* and *Prevotella intermedia*.

In this study, the CFS of all the thirty five (35) isolates was utilized to examine their antibacterial potential on the pathogens using the agar well diffusion method. Among the thirty-five (35) isolates, six (6) isolates showed antibacterial activities. The Chi-square test results (in Table 3 through 5) established that there was a significant and moderate association of 51.2 % (Phi value in Table 5). This means that all the inhibitions significantly dependent on the isolate and there was significant correlation of 51.2 % between the inhibitions and the isolates.

Obtained results reveled; the antagonist isolates were rod-shaped, gram-positive, catalase-producing and starch-hydrolyzing isolates. These morphological characteristics of soil isolates were similar to the characteristics reported by [52] from soil bacteria in Ngere tea catchment area of Murang’a County, Kenya. Most of the soil isolates were also rod-shaped, gram-positive, catalase-producing, starch-hydrolyzing, oxidase and phosphate-producing isolates. These characteristics in this present study are not sufficient to identify the isolates to the genus level [53].

This phenotypic method of identification is not reliable due to different phenotypic characteristics observed in bacteria of similar genera but of different species [54]. Molecular identification is more reliable and accurate for organism identification. Conserved regions within the 16S rRNA gene of the microbes’ DNA are usually utilized, which enhance sequence comparison. These provide an essential tool for the study evolutionary phylogenetic and molecular diversity [55]. Therefore, the antagonists isolates were subjected to molecular analysis for identification. Their DNA size was around 850 bp in length when subjected to Electrophoresis. According to [56], DNA size usually utilized for sequencing and comparison ranges from 500 bp to1500 bp. Therefore the utilization of 850 bp DNA size is suitable for sequencing and comparison.

The isolates’ 16S rRNA genes were sequenced for sequence comparison and evolutionary study. Blast analysis of the DNA sequences aligned Is 5, 8, 9, 10, 27 and 35 with 16S rRNA sequence of *Bacillus* sp. strain BCN2, *Brochothrix thermosphacta* strain P30C4, *Bacillus aryabhattai* strain KNUC205, *Alcaligenes faecalis* strain KEM24*, Bacillus arsenicus* strain CSD05 and *Lysinibacillus sphaericus* strain PRE16 with the similarity percentage of 82 %, 86 %, 93 %, 99 %, 83 % and 99 % and Gen Bank accession number JX045721.1, MH000378.1, JN051485.1, MK595710.1, HM100220.1 and EU880531.1 respectively. The dominating bacteria species are the *Bacillus* species. Phylogenetic analysis revealed Is 5 (B2) and ls 27 (H34) to form clade with *Bacillus* sp. BCN2 and *Bacillus arsenicus* strain CSD05 respectively. Forming a clade indicate that they are closely related having similar DNA sequences. Strains forming clade with other strains are closely related than with strains of other clades [57].

According to [58], these *Bacillus* species are predominantly soil bacteria, though they can be found in other habitats. [59] stated that their presence in soil in vast numbers is as a result of their ability to form resistance endospores and produce bioactive compounds which facilitate their resistance to fluctuating environmental conditions.

Vast numbers of research findings utilise 16S rRNA gene to identify these bacteria due to its high level of accuracy for identification. [60,61], identified *Brochothrix* species and *Bacillus* species respectively, using their 16S rRNA genes, all of which exhibit antagonizing activities. In a research study carried out by Rafiq et al. [62], an isolate from Passu glacier in Pakistan indicated as HTP6 was identified as *Alcaligenes faecalis* HTP6 by sequencing its 16S rRNA gene. *Lysinibacillus* species was also identified in a study carried out by [49] and [63] by sequencing similar genes.

The biochemical characteristics of *Bacillus* species in this present study, which include gram-positive, catalase-positive, urease-negative and starch hydrolyser were similar to the characteristics reported by [[64],48]. In their research study on the identification of *Bacillus* species, most *Bacillus* species isolated were gram-positive, catalase-positive, and unable to produce urease and starch-hydrolyzing bacteria.

*Brochothrix* species are commonly identified as food spoilage bacteria isolated from a wide range of animal food. Their presence in the soil as reported in this present study confirms other habitat such as soil they can be isolated for study. Their biochemical characteristics which include gram-positive, catalase-positive, urea-negative and inability to hydrolyse starch conform to the report of [65]. He reported their ability to produce catalase enzymes. They are gram-positive and cannot utilise exogenous urea.

Gram-positive, catalase-positive, urea-positive and inability to hydrolyse starch are the biochemical characteristics of *Lysinibacillus sphaericus* reported in the study. These characteristics were also reported by [26].

In this study, *in vitro* antibacterial activity screening of 35 isolates, only 6 isolates were able to exhibit antibacterial activity with varying percentage of antagonism. *Lysinibacillus sphearicus* strain PRE16 displayed highest antibacterial activities with 50 % antagonism percentage. It inhibits the growth of *Bacillus subtilis, Staphylococcus aureus* and *Escherichia coli* with zones of inhibition 23.00 ± 2.00, 18.00 ± 2.00 and 20.00 ± 4.00 respectively. [66], also reported the inhibition of *Bacillus subtilis, Staphylococcus aureus* and *Escherichia coli* by *Lysinibacillus sphearicus* with a zone of inhibition of 20 mm, 21 mm and 19 mm respectively.

*Bacillus* sp. BCN2, *Bacillus arsenicus* strain CSD05, *Bacillus aryabhattai* strain KNUC205, *Brochothrix thermosphacta* strain P30C4 and *Alcaligenes faecalis* strain KEM24 displayed antibacterial activity against *Pseudomonas aeruginosa* Figure Plate 3, with a zone of inhibition 15.00 ± 1.00, 18.00 ± 2.00, 15.30 ± 1.15, 11.00 ± 1.73 and 10.00 ± 1.15 respectively. [23], reported the antibacterial activity of a strain of *Bacillus*, *Bacillus* sp. strain FAS against *Pseudomonas aeruginosa, Staphylococcus aureus, Staphylococcus epidermidis, Escherichia coli* and *Klebsiella pneumonia* with zones of inhibition ranging from 17 to 27 mm. *Escherichia coli* was also susceptible to the inhibitory activity of *Bacillus arsenicus* strain CSD05 with a zone of inhibition of 17 mm. These are similar to the results obtained in this study. In the research study carried out by [67], they observed antibiofilm activity of *B. arsenicus* and other *Bacillus* species which include *B. pumilus* and *B. indicus* against biofilms of *P. aeruginosa* PA0I.

According to [6,47,66,[68], [69], [70]] the antibacterial activities displayed by the antagonizing bacteria are as a result of production of antibacterial agents Surfactin and bacteriocin which include mersacidin and erisin are some of the antibacterial compounds produced by *Bacillus* species responsible for their antibacterial activities [23,[71], [72], [73], [74]]. Brochocin-C is a bacteriocin produced by *Brochothrix* species, known for its broad spectrum activity [68].

[62], reported two antibacterial compounds produced by *Alcaligenes* species, namely kalimantacin and tunicamycin. These compounds were reported active against a vast number of pathogenic species, including the antibiotic resistance species. *Lysinnibacillus* species have been observed to produce siderophores and biosurfactants which are cell destructing metabolites [46]. They have been utilized for the production of silver nanoparticles, which exhibit antimicrobial activity due to the possession of a crystallographic surface structure and large surface to volume ratios [49,66].

## 5 Conclusion

This study demonstrated that farmland is a potential habitat for the isolation of bacteria with antibacterial activity against the growth of bacteria known for antibiotic resistance. *Lysinibacillus sphaericus* strain PRE16 displays highest antibacterial activity, inhibiting the growth of *Bacillus subtilis, Staphylococcus aureus* and *Escherichia coli*. *Pseudomonas aeruginosa* was susceptible to the antibacterial activity of *Bacillus* species, *Brochothrix thermosphacta* and *Alcaligenes faecalis.* These bacteria species could be a potential disease-control agent in eradicating most antibiotic resistance bacteria species. Further research includes characterizing the antibacterial agents responsible for the antagonizing activity, evaluating their activities and screening their expressions in different conditions. Development of effective formulation and techniques is also needed to improve the antibacterial effect of these bacteria.

## Supporting material

DNA sequence for the six antagonistic microorganisms namely: *Bacillus* sp. BCN2, *Brochothrix thermosphacta* strain P30C4, *Bacillus aryabhattai* strain KNUC205, *Alcaligenes faecalis* strain KEM24*, Bacillus arsenicus* strain CSD05 and *Lysinibacillus sphaericus* strain PRE16.

## Author statement

This is to certify that all the comment made by the reviewers has been carefully corrected as suggested.

We wish to state that the manuscript has not been submitted or presented for publication elsewhere.

Kindly consider it for publication in your highly esteem journal of Biocatalysis and Agricultural Biotechnology.

## Declaration of Competing Interest

The authors report no declarations of interest.
